# Refining Sentinel Lymph Node Biopsy Decisions for Clinically Node‐Negative Microinvasive DCIS

**DOI:** 10.1155/tbj/5479226

**Published:** 2026-05-18

**Authors:** Cien Huang, Priyanka Parmar, Noor Habboosh, Fardeen Bhimani, Yu Chen, David Entenberg, Maja Oktay, Anjuli Gupta, Jessica Pastoriza, Maureen McEvoy, Sheldon Feldman

**Affiliations:** ^1^ Albert Einstein College of Medicine, Bronx, New York, New York, USA, yu.edu; ^2^ Department of Surgery, Montefiore Medical Center, Bronx, New York, New York, USA, montefiore.org; ^3^ Department of Breast Surgery, Montefiore Einstein Comprehensive Cancer Center, Bronx, New York, New York, USA; ^4^ Department of Pathology, Albert Einstein College of Medicine, Bronx, New York, New York, USA, yu.edu

## Abstract

**Background:**

Ductal carcinoma in situ with microinvasion (DCISM), defined as invasion ≤ 1 mm, occurs in 5%–10% of DCIS cases. There is ongoing debate on whether DCISM should be managed as DCIS or as small invasive cancer. As treatments increasingly emphasize minimizing morbidity, efforts have focused on identifying low‐risk patients who may safely forgo axillary surgery, including sentinel lymph node biopsy (SLNB). However, axillary management of DCISM remains unclear due to limited data. This study aims to identify preoperative predictors of upstaging to invasive carcinoma on final pathology to guide SLNB use.

**Methods:**

A retrospective chart review was conducted of women aged ≥ 19 years with confirmed or suspected DCISM on initial biopsy who were clinically node negative and treated at a tertiary center from 2013 to 2023. Fisher’s exact test and Student′s *t*‐test were used for categorical and continuous variables, respectively. Univariate and multivariate analyses were performed to identify predictors of upstaging.

**Results:**

A total of 61 women with DCISM on initial biopsy were identified. Of these, 40.9% (25/61) were upstaged to invasive carcinoma on final pathology. Among upstaged patients, 95% (19/20) underwent SLNB at the time of surgery, and 12.5% (3/24) had positive lymph nodes. Time from biopsy to surgery did not differ significantly between groups (*p* = 0.12). Preoperative predictors of upstaging included palpable mass (OR 9.0, 95% CI 2.38–44.98, *p* = 0.003), mass on mammogram (OR 12.86, 95% CI 1.95–255.34, *p* = 0.02), and mass with calcifications (OR 13.34, 95% CI 1.30–255.08, *p* = 0.047).

**Conclusion:**

Clinically node‐negative DCISM patients without a palpable or mammographic mass (with or without calcifications) had a low risk of upstaging and lymph node involvement, suggesting that they may safely forgo SLNB. Identifying preoperative variables can guide risk stratifications and de‐escalation of axillary surgery. Larger prospective studies are needed to further inform guidelines for DCISM management in diverse populations.

## 1. Introduction

The diagnosis of early‐stage breast cancer, including ductal carcinoma in situ with microinvasion (DCISM), has increased with improved screening. DCISM, defined as invasion ≤ 1 mm in size, occurs in 5%–10% of DCIS cases, but its optimal management remains debated. While it shares features similar to DCIS and small invasive disease, there is no clear consensus on whether it should be treated similarly to DCIS or more aggressively. Although current guidelines recommend similar treatments to DCIS, the risk of upstaging to invasive disease and worse outcomes in certain populations, particularly Black patients, raises questions about the adequacy of these recommendations.

Compared to DCIS, DCISM is more likely to be ER‐/PR‐/HER2+ and to have aggressive pathology such as necrosis and high nuclear grade [1–3]. A meta‐analysis demonstrated that DCISM patients have significantly shorter disease‐free survival and locoregional recurrence‐free survival [4]. However, factors predicting DCISM upstaging to invasive ductal carcinoma (IDC) remain poorly defined, especially in diverse populations. Black race has been identified as an independent prognostic factor for worse survival in breast cancer, yet data on how clinicopathologic features influence DCISM upstaging across racial groups are lacking [2, 5]. To the best of our knowledge, this is the first study to evaluate preoperative predictors of DCISM upstaging to IDC in a racially diverse cohort.

The role of sentinel lymph node biopsy (SLNB) in DCISM also remains controversial. While SLNB can detect nodal disease, it carries the risk of axillary seromas, paresthesia, and upper extremity lymphedema, although these complication rates are low [6]. A study utilizing the National Cancer Database found that among 2609 DCISM patients undergoing SLNB, only 2.9% had nodal metastases. Low/intermediate‐grade tumors were associated with lower rates of SLN metastasis, which brings up the question of the utility of SLNB in the treatment for this subgroup of breast cancer patients [7].

As treatment shifts toward precision medicine and reducing morbidity, better risk stratification is needed to identify patients who may safely avoid SLNB. The objective of this study is to identify preoperative predictors of DCISM upstaging, with a focus on stratifying patients into low‐ and high‐risk groups for tailored management in a predominantly Black and Hispanic population.

## 2. Methods

### 2.1. Study Design

A retrospective chart review was conducted including women aged ≥ 19 years treated at Montefiore/Einstein Comprehensive Cancer Center from 2013 to 2023 who had DCIS with confirmed or suspected microinvasion on initial breast biopsy and were clinically node negative. All female patients who received testing, treatment, and therapies at Montefiore were eligible.

Patients with incomplete data variables, duplicates, no microinvasive disease, or concurrent invasive disease were excluded. The final size was determined after including patients from 2013 to 2023 who met the eligibility criteria mentioned above and excluding those who met the exclusion criteria.

The study was approved by the Montefiore Institutional Review Board, and patient confidentiality was maintained per institutional and HIPAA regulations. Data were retrieved from electronic medical records, and patient identifiers were deidentified. Data access was limited only to the authors listed in this study.

### 2.2. Outcomes

The primary outcome was defined as upstaging to invasive cancer on final surgical pathology.

### 2.3. Statistical Analysis

Descriptive statistics were used to summarize the demographic characteristics of the study population. Continuous variables were reported as means with standard deviations, while categorical variables were reported as frequencies and percentages. Fisher’s exact test was used for categorical variables, and Student′s *t*‐test was used for continuous variables. Univariate analysis was conducted to assess the association between individual preoperative variables and the likelihood of upstaging to invasive carcinoma. To identify independent predictors of upstaging, multivariate logistic regression analysis was performed. Variables significant in the univariate analysis were included in the multivariable model, along with clinically relevant factors such as biopsy technique and hormone receptor status. Results were expressed as odds ratios (ORs) with 95% confidence intervals (CIs). Mammogram (MG) and MRI mass size cutoff was calculated by separating control mass sizes into tertiles and using the 66th percentile as the cutoff number.

All statistical analysis was performed using RStudio Version 2024.04.2 + 764, with *p* value < 0.05 considered statistically significant.

## 3. Results

**FIGURE 1:**
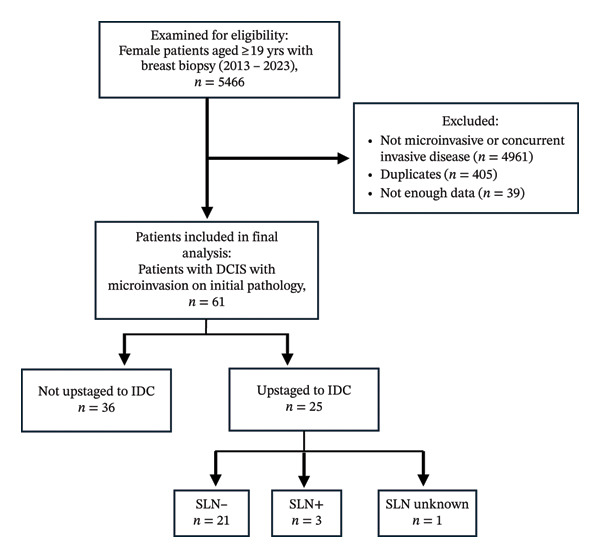
Study population.

A total of 5466 women met the inclusion criteria of aged 19 years or older with breast biopsy performed from 2013 to 2023. Subsequently, 4961 patients were excluded as they did not have microinvasive DCIS or had concurrent invasive disease, 405 duplicates were excluded, and 39 patients were excluded due to missing data (Figure 1).

A total of 61 women met the inclusion criteria and were included in the final study (Figure 1). Among them, 40.9% (*n* = 25/61) were upstaged to invasive carcinoma on final pathology. Of the entire cohort, 47.5% (*n* = 29/61) of patients were White and 42.6% (*n* = 26/61) were Black, with 39.3% (*n* = 24/61) Hispanic. Demographics including age, race, ethnicity, and insurance coverage between patients that upstaged vs. did not upstage were similarly distributed (Table 1).

**TABLE 1:** Baseline characteristics of study population.

Demographic variable	Not upstaged (*n* = 36)	Upstaged (*n* = 25)	*p* value
Mean age (SD)	58.4 (10.03)	56.9 (9.10)	0 47
Race			
Caucasian	15 (41.7%)	14 (56%)	0.34
Black	16 (44.4%)	10 (40.0%)	
Other	5 (13.9%)	1 (4.0%)	
Ethnicity			
Hispanic	13 (36.1%)	11 (44.0%)	0.54
Non‐Hispanic	23 (63.9%)	14 (56.0%)	
Insurance			
Medicaid/Medicare	14 (38.9%)	12 (48.0%)	0.55
Medicaid/Medicare + supplemental	4 (11.1%)	1 (4.0%)	
Private	18 (50.0%)	12 (48.0%)	

Comparing preoperative characteristics between the two groups, MG size between patients who were not upstaged (mean = 20.6 mm, SD = 15.4 mm) and upstaged patients (mean = 36.7 mm, SD = 36.5) was found to be significantly different (*p* = 0.033) (Table 2). Similarly, there was a significant difference in the presence of a clinically palpable mass between the two groups (*p* = 0.0028), and mass and/or calcifications on MG (*p* = 0.0029) (Table 2). Grade, hormone receptor status, HER2 status, biopsy needle gauge, number of biopsy cores, and MRI mass size were not statistically significant between upstaged vs. not upstaged patients (Table 2).

**TABLE 2:** Preoperative clinicopathologic features.

Preoperative variable	Not upstaged (*n* = 36)	Upstaged (*n* = 25)	*p* value
Grade			
Low‐intermediate	14 (41.2%)	8 (33.3%)	0.54
High	20 (58.8%)	16 (66.7%)	
HR status			
HR positive	27 (75.0%)	15 (60.0%)	0.21
HR negative	9 (25.0%)	10 (40.0%)	
HER2 status			
HER2 positive	4 (50.0%)	2 (25.0%)	0.30
HER2 negative	4 (50.0%)	6 (75.0%)	
Mammogram size			
Mean in (mm) (SD)	20.6 (15.4)	36.7 (36.5)	0.033
Palpability of mass			
Not palpable	32 (94.1%)	13 (54.2%)	0.0028
Palpable	2 (5.9%)	11 (45.8%)	
Findings on mammogram			
Calcification	30 (90.9%)	14 (58.3%)	0.0029
Mass	1 (3.0%)	6 (25.0%)	
Calcification and mass	2 (6.1%)	4 (16.7%)	
Biopsy gauge			
Mean (SD)	9.9 (1.9)	10.9 (2.4)	0.14
Number of biopsy cores			
Mean (SD)	6.2 (2.8)	7.6 (4.4)	0.19
MRI size			
Mean in (mm) (SD)	63 (90.0)	69.2 (37.0)	0.85

Abbreviation: HR, hormone receptor.

Preoperative variables in the univariate analysis that predicted higher odds of upstaging included palpable mass (OR 9.03, 95% CI 2.38–44.98, *p* = 0.003) and the finding of mass on MG (OR 12.86, 95% CI 1.95–255.34, *p* = 0.02) (Table 3). We then performed multivariate analysis adjusting for key clinicopathologic and preoperative variables in multiple models given our sample size limitations (Table 4). Multivariate analysis was performed with adjustment for palpable mass, mass on MG, mass with calcifications on MG, mass size ≥ 24 mm, and private insurance in different combinations.

**TABLE 3:** Univariate regression analysis of preoperative variables.

Preoperative variable	OR (95% CI)	*p* value
Age ≥ 65 yrs (ref: age < 65 yrs)	0.65 (0.18–2.14)	0.5
Black race (ref: non‐Black)	0.83 (0.29–2.34)	0.7
Hispanic (ref: non‐Hispanic)	1.39 (0.49–3.98)	0.5
Private insurance (ref: Medicaid/Medicare)	0.92 (0.33–2.57)	0.9
High grade (ref: low‐intermediate)	1.40 (0.48–4.28)	0.5
HR negative (ref: HR positive)	2.00 (0.67–6.13)	0.2
Palpable mass (ref: nonpalpable mass)	9.03 (2.38–44.98)	0.003
Mass on MG (ref: calcs on MG)	12.86 (1.95–255.34)	0.02
Mass and calcs on MG (ref: calcs on MG)	4.29 (0.75–33.67)	0.12
MG size ≥ 24 mm (ref: MG size < 24 mm)	2.36 (0.79–7.33)	0.1
MRI size ≥ 48 mm (ref: MRI size < 48 mm)	2.50 (0.31–25.92)	0.4
Biopsy gauge of 9 (ref: gauge > 9)	0.41 (0.11–1.41)	0.2
≥ 4 biopsy cores (ref: < 4 biopsy cores)	0.87 (0.17–4.94)	0.9

*Note:* MG: mammogram.

Abbreviation: HR, hormone receptor.

**TABLE 4:** Final pathologic characteristics of upstaged vs. not upstaged patients.

Pathologic variable	Not upstaged (*n* = 36)	Upstaged (*n* = 25)	*p* value
Grade			
Low‐intermediate	12 (35.3%)	6 (26.1%)	0.79
High	22 (64.7%)	17 (73.9%)	
Comedonecrosis			
Frequency	21/30 (70%)	11/18 (61.1%)	0.53
EIC			
Frequency	7/17 (41.2%)	9/15 (60.0%)	0.29
Final hormone status			
HR positive	27 (84.4%)	14 (63.6%)	0.080
HR negative	5 (15.6%)	8 (36.4%)	
Type of surgery			
Lumpectomy	28 (77.8%)	13 (52.0%)	0.035
Mastectomy	8 (22.2%)	12 (48.0%)	
Days from biopsy to surgery			
Mean (SD)	47.6 (25.6)	61.8 (43.5)	0.12
Number undergoing axillary surgery			
Frequency (%)	20/36 (55.6%)	20/25 (80.0%)	0.030
Type of axillary surgery			
SLNB	20/20 (100%)	19/20 (95%)	0.99
ALND	0/20 (0%)	1/20 (4%)	
Timing of axillary surgery			
Same time as initial surgery	20/20 (100%)	19/20 (95%)	0.99
Later than initial surgery	0 (0%)	1/20 (4%)	
Number of nodes taken			
Mean (SD)	2.5 (1.1)	2.6 (1.5)	0.74
Node result			
Positive	0 (0%)	3 (12.5%)	0.10
Negative	20 (100%)	21 (87.5%)	

Abbreviation: EIC, extensive intraductal component.

Multivariate analysis Model A was adjusted for palpable mass, mass on MG, and mass with calcifications on MG. In this model, no variable was found to be a statistically significant predictor (Model A of Table S1). Next, multivariate analysis Model B was adjusted for mass on MG, mass with calcifications on MG, and mass size ≥ 24 mm. Only palpable mass was a significant predictor in this model (OR 13.74, 95% CI 2.38–129.12, *p* = 0.008; Model B of Table S1). The third multivariate analysis model (Model C) adjusted for palpable mass, MG size, and MG size ≥ 24 mm. This model demonstrated that mass on MG (OR 56.61, 95% CI 4.88–1735.98, *p* = 0.005) and mass with calcifications on MG (OR 13.43, 95% CI 1.30–255.08, *p* = 0.047) were significant predictors of upstaging (Model C of Table 4). The final multivariate model (Model D of Table S1) with adjustment for private insurance showed that high grade (OR 18.96, 95% CI 1.54–643.68, *p* = 0.044), mass on MG (OR 536.23, 95% CI 14.93–81,312.29, *p* = 0.003), mass and calcifications on MG (OR 72.49, 95% CI 1.70–12,844.55, *p* = 0.048), and MG size ≥ 24 mm (OR 14.11, 95% CI 1.76–275.99, *p* = 0.031) were significantly associated with increased odds of upstaging.

Lastly, an analysis of the final surgery and pathology was conducted. There was a difference in the distribution of lumpectomy vs. mastectomy between the two groups of patients with a higher number of not upstaged patients undergoing lumpectomy and more upstaged patients undergoing mastectomy (*p* = 0.035) (Table 4). Of note, upstaged patients more frequently underwent axillary surgery (80.0% vs. 55.6%, *p* = 0.030). Only 5% (*n* = 1/20) had axillary surgery after upstaging was confirmed while the rest of the patients had axillary surgery at the time of their breast surgery with no statistical significance between upstaged vs. not upstaged patients in terms of the timing of axillary surgery (*p* = 0.99). There was no difference between the two groups of patients in terms of days from biopsy to surgery (*p* = 0.12) or number of lymph nodes taken (*p* = 0.74). In terms of final pathology, there were no differences in grade (*p* = 0.79), comedonecrosis (*p* = 0.53), extensive intraductal component (EIC) (*p* = 0.29), final hormone receptor status *p* = 0.080), or lymph node status (*p* = 0.10) (Table 4).

## 4. Discussion

In our racially diverse patient cohort where 42.6% were Black patients and 39.3% were Hispanic, 40.9% of DCISM cases were upstaged to IDC, which is similar to previous studies reporting around 30% of upstaging [8]. In both the univariate and multivariate models, palpable mass was found to be significantly associated with upstaging to IDC on final pathology. These findings corroborated the Weaver et al. study that DCISM presenting with a more substantial or clinically detectable lesion may carry a higher risk of harboring invasive components that are not detected on initial biopsy given its larger size and random distribution of invasive components in a large, heterogenous mass [9, 10]. There is discordance in current literature with some studies demonstrating that clinically palpable mass on examination is associated with DCISM upstaging, while others showing no significant predictive values, including palpable mass or tumor architectural distortion, were found to inform the need for SLNB in DCISM patients [8, 11, 12]. However, the patient racial makeup of these studies was not specified or have low percentages of Black patients (17.1% in the Phantana‐Angkool et al. study), which further highlight the importance of the significance of mass palpability in our racially diverse patient population [8, 13].

MG findings of mass and mass with calcifications were demonstrated to be significant predictors of upstaging in the multivariate model of this study. Of note, given the wide range in the CI, the effect size of the OR should be interpreted with caution as this is likely due to the small sample size. Interestingly, previous studies showed that masses or masses with calcifications on MG were due to invasive cancers, and calcifications on MG were most likely to be associated with DCIS and more rapidly growing disease [14, 15]. This may be a reflection of the tumor biology of more aggressive DCIS with higher grade such as DCISM [16].

There is mixed evidence with some studies showing hormone receptor negativity to be associated with upstaging and others showing that HER2 positivity is a predictor [7, 17]. A recent Italian multicenter study by Vanni et al. demonstrated that multifocal lesions and hormone receptor negativity were associated with sentinel lymph node metastasis [13]. Our univariate and multivariate analyses demonstrated that hormone receptor status was not associated with upstaging. This deviated from existing literature that demonstrated that when compared to invasive disease, DCISM is less likely to be ER+/PR+ and more likely to be HER2+ [2]. In our study, HER2 status was analyzed as a preoperative variable, but the sample size was much smaller compared to other variables given that not all biopsy samples were adequately large enough for HER2 analysis (*n* = 8 in not upstaged patients and *n* = 8 in upstaged patients); therefore, we cannot make any significant conclusions on HER2 status on DCISM upstaging.

In determining the utility of SLNB in patients with DCISM, it is important to consider the difference in prognosis between DCISM and pure DCIS. A meta‐analysis of 26 studies demonstrated that disease‐free survival and locoregional recurrence‐free survival were significantly shorter in patients with DCISM [4]. Factors that contribute to this observed outcome include larger lesions, axillary lymph node metastasis, comedonecrosis, or hormone receptor negativity/HER2 positivity, which are indications of more aggressive biology [4, 18]. Although DCISM may have more aggressive biology, guidelines generally recommend treating it like DCIS because each lesion likely comprises mostly DCIS, and treatments would reflect traditional DCIS pathways. The 2026 joint SSO‐ASTRO‐ASCO guidelines recommend treating DCISM with breast‐conserving surgery (BCS) with whole‐breast radiation [19]. The 2026 NCCN guidelines similarly advise the following DCIS pathways: BCS without lymph node surgery or mastectomy with SLNB. Radiation is recommended after BCS, though some low‐risk patients may omit it, and endocrine therapy depends on menopausal status and treatment type [20]. Chemotherapy and HER2‐specific therapy are not routinely recommended for DCISM patients.

Despite similar treatment approaches, indications for SLNB in DCISM remain unclear as current guidelines do not concur on whether SLNB is necessary in this subset of patients [11]. NCCN recommends SLNB mainly for mastectomy, procedures that may compromise future SLNB, or oncoplastic BCS [20]. The 2025 ASCO guidelines advise against SLNB with BCS, recommending it primarily for mastectomy or selected patients (e.g., obese, male, pregnant, or prior breast/axillary surgery) [21]. A retrospective study using the National Cancer Database showed that SLNB is performed more often with mastectomy, with a higher nodal positivity rate, which may reflect the preoperative factors that led to the more aggressive clinical decision of mastectomy in the first place [11, 22]. This is also demonstrated in our study in which there is a significantly higher percentage of patients undergoing mastectomy who were upstaged compared to those with BCS.

In our cohort, 97.5% (*n* = 39/40) of patients had axillary surgery at the time of breast surgery. A prospective study conducted in England from 2003 to 2012 showed that axillary surgery was more frequently performed in those with DCISM (60.4%) [11]. Our study showed similar rates of DCISM patients undergoing axillary surgery (65.5%, *n* = 40/61), with 80.0% (*n* = 20/25) of those who were upstaged receiving the surgery. In our study, of those who received axillary surgery, 12.5% had positive lymph nodes, which is similar to previous studies where nodal metastasis rates ranged from 2.9% to 9.2% for patients with DCISM [7, 8, 11, 22, 23]. In addition, of the patients who received axillary surgery, the majority received SLNB as opposed to axillary lymph node surgery (100% in not upstaged patients and 95% in upstaged patients).

Choosing patients for de‐escalation of SLNB has become important given the side effects associated with axillary surgery. The Choosing Wisely recommendations by the Society of Surgical Oncology in 2016 recommended against the use of SLNB in early stage, hormone receptor positive breast cancer patients who are aged 70 years and older [24]. Of note, this must be taken into consideration as this study’s patient population was diagnosed from 2013 to 2023. However, the mean age of patients who were not upstaged (58.4 years, SD = 10.3) and upstaged patients (56.9 years, SD = 9.10) was not statistically different (*p* = 0.47), and the majority of patients were younger than age 70. Therefore, many patients did not meet the Choosing Wisely guidelines and would be recommended to have SLNB. Further analysis with respect to age and SLNB rates in the two groups would be prudent to further investigate whether the implementation of the Choosing Wisely guidelines affected the rate of axillary surgery in these two groups and affected the rate of upstaging.

More recent randomized controlled trials including the SOUND trial found that omission of SLNB in early‐stage invasive breast cancer patients with tumors ≤ 2 cm and are clinically node‐negative was not inferior to those who received SLNB [25]. Similarly, the INSEMA trial found that omission of SLNB in clinically node‐negative, T1/T2 invasive breast cancer with lesions ≤ 5 cm was noninferior to SLNB [26]. In accordance with the findings of the SOUND and INSEMA trials, the 2026 NCCN guidelines recommend that patients > 50 years and postmenopausal who are cT1N0, node negative by axillary ultrasound, HR+/HER2‐, with Grade 1–2 tumors and those who receive whole‐breast RT and endocrine therapy may be considered for omission of SLNB [20]. It is important to note that only patients undergoing BCS with radiotherapy were included in the SOUND trial, which brings up the question of the utility of SLNB in DCISM patients who are clinically advised to undergo mastectomy [25]. Similarly, only patients undergoing BCS were included in the INSEMA trial [26]. In addition, patients with DCISM (T1mi) represent a subgroup that has a better prognosis than higher grade tumors but only comprises a small percentage of the study population in both the SOUND and INSEMA trials, 9.4% and 0.8%, respectively. Finally, the patient population of the two trials was predominantly homogenous White in the INSEMA trial and unspecified in the SOUND trial, which differs from our study in which 42.6% are Black and 39.3% Hispanic. This highlights the need for larger studies including diverse patient populations. There may be utility in considering SLNB in initially DCISM patients upstaged to IDC on final pathology that do not adhere to the criteria of these two trials.

The final pathologic characteristics of patients who were not upstaged and upstaged patients were similar in terms of grade, comedonecrosis, EIC, and final hormone status. Interestingly, this delineated from current literature that demonstrated that DCISM has higher nuclear grade, comedonecrosis, and higher rates of hormone receptor negativity [27, 28].

Few studies investigate whether there is an association between race/ethnicity and DCISM upstaging. A study using the SEER database utilizing 3400 patients demonstrated that, when compared to non‐Hispanic whites (83.3%), non‐Hispanic Blacks (16.7%) were more likely to have higher grade disease but no difference in the molecular subtype. Non‐Hispanic Blacks were also more likely to undergo more aggressive treatment with axillary lymph node dissection and chemotherapy and have worse outcomes than non‐Hispanic Whites [5]. In our study, 42.6% of the study population were Black, a much higher percentage than the previous study [5]. A large 2025 National Cancer Database study utilizing patients diagnosed between 2012 and 2019 by Lava et al. showed that Black race was an independent predictor of nodal positivity [22]. Although none of the demographic variables, including race, examined in this study were demonstrated to be significantly associated with increased odds of upstaging, the high proportion of Black patients in this current study should be taken into account given that previous studies have shown that Black patients have higher rates of nodal disease, which may be attributed to a combination of socioeconomic status, systemic discrimination, and more unfavorable tumor genomics [5, 29]. In addition, in our study population, 50.0% of patients who were not upstaged and 52% of upstaged patients were covered by public insurance (Medicaid/Medicare), demonstrating the diverse socioeconomic status of this study population. Further studies with larger sample sizes are needed to determine if there is a racial or socioeconomic association with DCISM upstaging.

Our study has a few strengths. To our knowledge, this is the first study to identify preoperative characteristics associated with upstaging of DCISM to IDC on final pathology in a significantly large Black and Hispanic patient population. We do note limitations of our study including the retrospective study cohort, single institution cohort, and small sample size of 61 patients, which limits the generalizability of our findings. Additionally, there was a lack of delineation between suspected vs. definitive DCISM on initial biopsy given that cases were reviewed by different pathologists and there is inherent clinician diagnostic variability. Future studies should consider examining suspected vs. definitive DCISM on initial biopsy as a possible preoperative variable. However, to our knowledge, this is the first study conducted in a predominately Black and Hispanic patient cohort, a group that has been previously reported to have worse overall survival among breast cancer patients; therefore, our study is important in elucidating predictor variables for upstaging in a racially diverse population.

## 5. Conclusion

This study identified the following as significant preoperative predictors of DCISM upstaging to invasive cancer: palpable mass and mass with or without calcifications on mammogram. These findings support a more tailored approach to SLNB, focusing on patients at higher risk for invasive disease while sparing low‐risk patients from unnecessary axillary surgery. Future research should further validate these predictors and explore their implications for treatment guidelines including diverse populations.

## Author Contributions

Cien Huang: conceptualization, methodology, data curation, writing–original draft, and writing–review and editing. Priyanka Parmar: conceptualization, methodology, formal analysis, writing–original draft, writing–review and editing, and funding acquisition. Noor Habboosh, Fardeen Bhimani, Anjuli Gupta, and Jessica Pastoriza: conceptualization. Yu Chen: formal analysis. David Entenberg: conceptualization and resources. Maja Oktay: resources, supervision, and project administration. Maureen McEvoy: methodology and supervision. Sheldon Feldman: supervision and project administration. All authors: writing–review and editing.

## Funding

This work was supported by the National Institute of Health [T32 grant CA200561].

## Disclosure

The preliminary findings of this study were presented as a poster presentation at San Antonio Breast Cancer Symposium in December of 2024, and the abstract was subsequently published in AACR Clinical Cancer Research in June 2025. The published abstract can be found at the following: https://doi.org/10.1158/1557-3265.SABCS24-P5-12-12.

## Conflicts of Interest

The authors declare no conflicts of interest.

## Supporting Information

Additional supporting information can be found online in the Supporting Information section.

## Supporting information


**Supporting Information** Table S1 can be found in the supplemental section, which detailed the results of four multivariate regression analysis models of preoperative variables.

## Data Availability

The data that supports the findings of this study are available from the corresponding author upon reasonable request.
