# Supplementary material for: Refining Sentinel Lymph Node Biopsy Decisions for Clinically Node‐Negative Microinvasive DCIS
Source: Breast J. 2026 May 18;2026:5479226. doi: 10.1155/tbj/5479226 (PMC13181275; doi:10.1155/tbj/5479226)
Supplement: Supplementary file 1 — Supporting Information Table S1 can be found in the supplemental section, which detailed the results of four multivariate regression analysis models of preoperative variables. [file TBJ-2026-5479226-s001.docx]

**Table S1: Multivariate regression analysis of pre-operative variables**


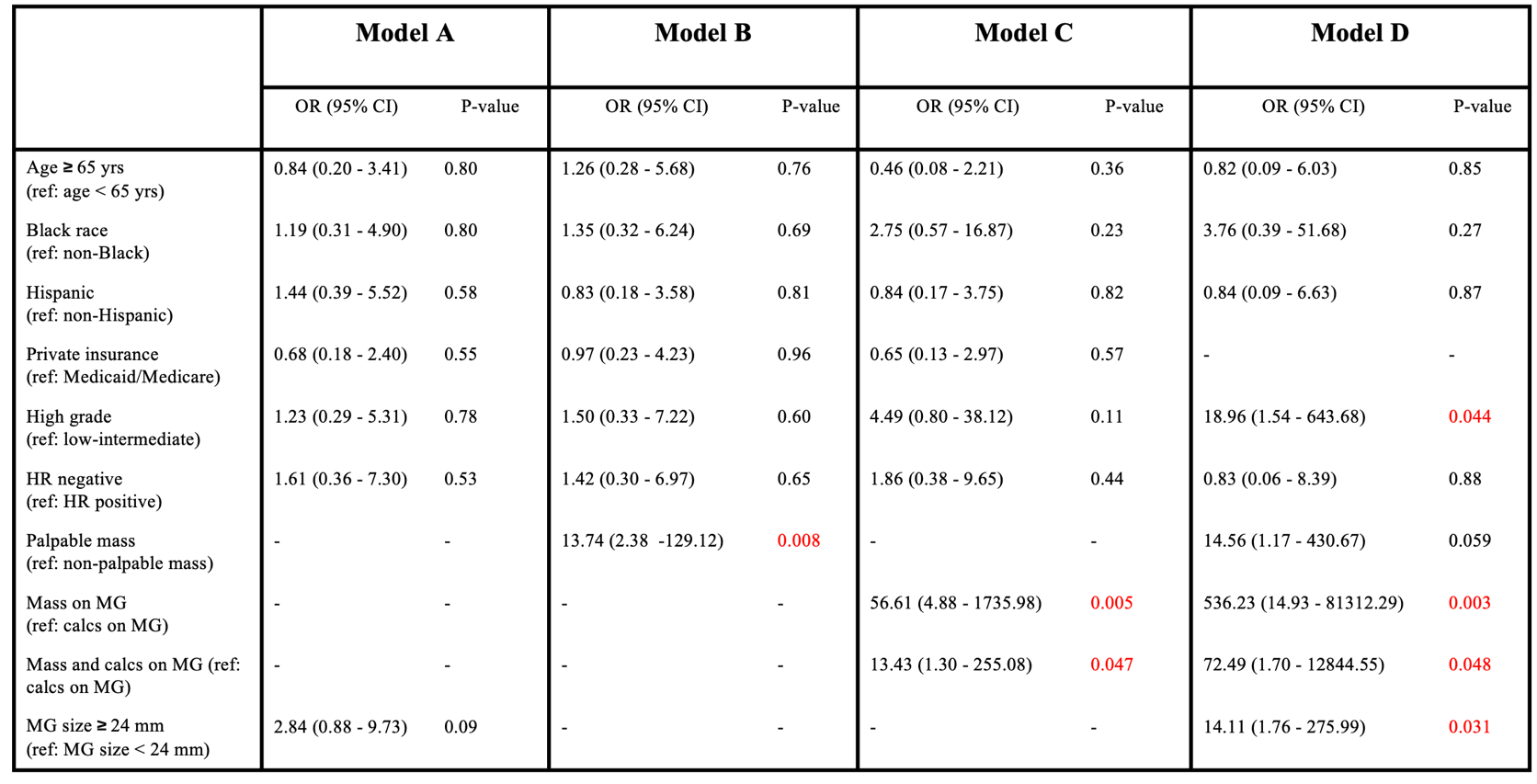
Multivariate Model A adjusted for palpable mass, mass on MG, and mass with calcifications on MG. Multivariate Model B adjusted for mass on MG, mass with calcifications on MG, and MG size ≥ 24 mm. In the third model, Model C, adjustment was made for palpable mass and MG size ≥ 24 mm. The final model (Model D) was adjusted for private insurance.

MG: mammogram

HR: hormone receptor
